# The effect of postoperative antibiotics in reducing complications following primary surgical closure of oroantral communications secondary to tooth extraction: a retrospective multicenter study

**DOI:** 10.1007/s00784-026-07189-9

**Published:** 2026-09-30

**Authors:** Melih M. Bakacak, Jean Pierre T.F. Ho, Erik H. van der Meij, Willem H. Schreuder, Johannes N. Lodders

**Affiliations:** 1 Department of Oral and Maxillofacial Surgery, Amsterdam University Medical Centers (UMC), University of Amsterdam, Amsterdam, Netherlands; 2 Academic Centre for Dentistry Amsterdam (ACTA), University of Amsterdam and Vrije Universiteit Amsterdam, Amsterdam, Netherlands; 3 Department of Oral and Maxillofacial Surgery, Northwest Clinics, Alkmaar, Netherlands

**Keywords:** Oroantral communication, Oroantral fistula, Tooth extraction, Antibiotic prophylaxis, Postoperative complications, Maxillary sinusitis

## Abstract

**Objectives:**

Maxillary canine, premolar and molar extractions may result in oroantral communication (OAC). Surgical closure aims to prevent the development of a persistent oroantral fistula (OAF), thereby reducing the risk of maxillary sinusitis. There is no consensus regarding the benefit of the use of perioperative antibiotics. This study evaluated the effect of postoperative antibiotics on complications after primary OAC closure following tooth extraction.

**Materials and methods:**

Eligible patients were identified by retrospectively reviewing the medical records of patients who underwent surgical closure of an OAC at Amsterdam UMC-VUmc and Amsterdam UMC-AMC from 2000 to 2025. Among these patients, the associations of various exposures, including postoperative antibiotic use, with overall complications, infectious complications, and persistent OAF were assessed. Group differences were assessed using the chi-square and independent t-tests, while univariate and multivariable associations were assessed using logistic regression (*p* < 0.05). Number needed to be exposed to benefit (NNEB) and harm (NNEH) were calculated.

**Results:**

A total of 725 potentially eligible participants were identified, of whom 505 were eligible; 308 (61.0%) were male, and 251 (49.7%) received postoperative antibiotics. Overall complications occurred in 11.7%, infectious complications in 8.2%, and persistent OAF in 3.6%. Postoperative antibiotic use was significantly associated with fewer overall and infectious complications and fewer persistent OAFs. (NNEB 6.9, 6.9, and 21.4, respectively). Autoimmune disease and use of intra-alveolar hemostatic agent were associated with increased overall complications. Soft tissue (partial) impaction was associated with increased risk for infectious complications. Use of intra-alveolar hemostatic agent and soft tissue (partial) impaction were associated with increased risk for persistent OAF.

**Conclusion:**

Postoperative antibiotic use after primary OAC closure following tooth extraction was associated with reduced risk of overall and infectious complications and persistent OAFs. Prospective studies are needed to confirm these findings and guide antibiotic use.

**Clinical relevance:**

Oral healthcare professionals should be aware of the potential benefit of postoperative antibiotics in preventing complications. Therefore antibiotic prophylaxis could be considered in patients with obvious risk factors or if an uneventful outcome is highly desirable.

## Introduction

The extraction of maxillary canines, premolars, and molars may result in an oroantral communication (OAC) because of their close anatomical relationship with the maxillary sinus [1]. Reported incidence rates of OAC following maxillary tooth extraction range from 5% to 13%, depending on the tooth location. [2, 3] Although incidence rates vary, OAC is frequently encountered due to the high number of maxillary tooth extractions performed in daily practice [2–4]. The primary treatment goal for OACs following tooth extraction is to restore the anatomical barrier between the oral cavity and the maxillary sinus, preventing complications such as acute and chronic sinusitis [4–7]. Additionally, a persistent oroantral fistula (OAF) can significantly impact a patient’s quality of life, leading to symptoms such as pain, swelling, and nasal regurgitation [8]. 

Various surgical techniques have been described for the closure of OACs. Among these, local pedicled flaps such as the trapezoidal flap, the palatal rotation flap and the buccal fat pad (Bichat’s) graft are the most widely accepted approaches [4–9]. In addition, small OACs are sometimes managed with absorbable hemostatic agents, particularly in general dental practice. The preferred method for the initial closure of an OAC following tooth extraction is determined by the surgeon’s clinical judgment, the size of the defect, and clinical circumstances [4, 10, 11]. 

At present, no international guidelines exist regarding the administration of peri-operative antibiotics after primary closure of an OAC following tooth extraction. According to the Dutch guideline, peri-operative antibiotic prophylaxis is not routinely recommended for the management of OAC following tooth extraction [12]. This recommendation is supported by limited evidence, including a single study reporting that antibiotic prophylaxis is not indicated prior to the surgical closure of an OAC [13]. In contrast, some authors advocate routine postoperative antibiotic use, although supporting evidence is scarce and is largely based on expert opinion [14–17]. To the authors’ knowledge, the existing literature does not provide evidence of a difference between preoperative and postoperative antibiotic administration in reducing complications after OAC closure. In light of this limited evidence and the potential adverse effects of antibiotic use, including allergic reactions and gastrointestinal side effects, as well as associated healthcare costs, critical evaluation of their use is warranted. Furthermore, the growing concern of antibiotic resistance in healthcare highlights the need to determine whether peri-operative antibiotics for OAC closure are supported by evidence [18–20]. 

Therefore, this study aimed to gain further insight into the association between postoperative antibiotic use and complications following primary surgical closure of an OAC after tooth extraction. Additionally, the study aimed to identify potential predictors of postoperative complications, including overall and infectious complications and persistent OAF. We hypothesized that postoperative antibiotic use would be associated with a reduced risk of postoperative complications, particularly infectious complications.

## Materials and methods

A retrospective cohort study was conducted in the Department of Oral and Maxillofacial Surgery at the Amsterdam University Medical Centers (Amsterdam UMC), The Netherlands. Two locations of the Amsterdam UMC were used for inclusions: VU University Medical Center (VUmc) and the Academic Medical Center (AMC), both tertiary teaching hospitals in Amsterdam.

Potentially eligible subjects were initially identified from the institutional database. Subsequently, medical records were manually reviewed to assess eligibility according to the predefined inclusion and exclusion criteria. Patients were included in this study if they underwent extraction of at least one maxillary tooth between January 2000 and March 2025 resulting in an OAC that was surgically closed. Cases involving extractions carried out in general dental practice and were referred to Amsterdam UMC for OAC closure were included. Only patients who underwent primary surgical closure of an OAC within 48 h were eligible for this study. Second-time closures of an OAC or patients with pre-existing maxillary sinusitis were excluded. If an OAC was caused by any procedure other than tooth extraction, the patient was excluded. Patients in whom closure was achieved using only a hemostatic agent, without primary closure, were excluded.

The study protocol was granted approval by the Institutional Review Board of Amsterdam University Medical Center (registration number: 2021.0668).

Patient related, radiographic and surgical data were obtained by retrospective reviewing patient records. Patient related data included patient age, sex, tobacco use, comorbidities, history of head and neck radiotherapy, and medication use. Radiographic data comprised the fraction of root overlapping the maxillary sinus (FROMS) on pre-extraction radiographs, as defined by Bakacak et al. [21] Panoramic and periapical radiographs were evaluated using imaging software (AGFA Enterprise Imaging XERO Viewer, version 8.0.1 Mortsel, Belgium). The following surgical variables were recorded: side, types (cuspid/premolar/molar), and indication for tooth extraction, hospital where the OAC was closed, site of the OAC (left or right), degree of soft-tissue impaction (completely erupted/partially erupted/non-erupted), timing of OAC closure, type of anesthesia, years of surgeon’s experience, presence of teeth adjacent to the OAC, occurrence of maxillary tuberosity fracture, closure technique, use of hemostatic agents, suture material, and postoperative medication, with particular emphasis on the prescription of antibiotics and types of antibiotics.

Within the subgroup of patients receiving postoperative antibiotics, an exploratory analysis was performed to assess whether type of antibiotic was associated with postoperative outcomes.

In all cases, in accordance with standard procedures, one of the following methods was used to diagnose an OAC immediately after tooth removal, depending on whether the tooth was removed under local or general anaesthesia. For the procedures under local anaesthesia, a Valsalva test was performed by instructing the patient to gently blow air through the nose while pinching the nostrils, with the clinician observing for air escaping through the extraction socket. This test was omitted if the patient was under general anaesthesia. Alternatively, a visual inspection was used to identify a direct defect in the alveolar bone and a visible opening in the Schneiderian membrane. In some cases, gentle blunt probing of the extraction socket was performed without applying pressure, to detect an OAC. Patients were instructed to avoid nose blowing, sneezing with a closed mouth, and any activities that may increase intranasal pressure for at least 14 days following diagnosis of an oroantral communication. In addition, patients were prescribed a soft diet and instructed to report immediately if symptoms such as nasal regurgitation, sinus discomfort, or signs of infection occurred.

Postoperative complications were defined as any deviation from the expected postoperative course requiring pharmacological treatment and/or surgical intervention. Complications were classified according to the Clavien–Dindo classification (Table 1) [22]. All patients received a follow-up approximately two weeks postoperatively in both hospitals as part of standard care. Patients without documented follow-up were excluded.

**Table 1:** Clavien–Dindo classification of complications

Grade	Definition	Mode of therapy
I	Any deviation from the normal postoperative course.	No pharmacological or surgical treatment, endoscopic or radiological intervention required. Acceptable treatments include antiemetics, antipyretics, analgesics, diuretics, electrolytes, and physiotherapy.
II	Normal postoperative course altered.	Pharmacological treatment other than those permitted for Grade I. Includes blood transfusions and total parenteral nutrition.
III	Complications requiring surgical, endoscopic, or radiological intervention.	IIIa: Intervention not under general anesthesia. IIIb: Intervention under general or epidural anesthesia.
IV	Life-threatening complications requiring ICU management.	IVa: Single organ dysfunction (including dialysis). IVb: Multi-organ dysfunction.
V	Death of a patient.	

*ICU* intensive care unit

SPSS Software package (version 28 IBM Inc., Chicago, IL, USA) was used for statistical analysis. The outcome measures used for statistical analysis were: (1) the overall complication rate; (2) infectious complications; and (3) failure of the OAC closure. Infectious complications included (intraoral) wound infection and maxillary sinusitis. Odontogenic maxillary sinusitis was defined in accordance with the criteria outlined in the European Position Paper on Rhinosinusitis and Nasal Polyps (EPOS) [23]: the presence of at least one symptom—either nasal obstruction or discolored nasal discharge—with or without accompanying facial pain or pressure and/or a reduction or loss of olfactory function. The complaints should be lateralized to the operated side and should be confirmed with a (cone beam) CT scan. Failure of OAC closure was defined as the development of a persistent oroantral fistula (OAF).

Descriptive statistics were used to summarize and classify all patient characteristics by postoperative antibiotic use. Differences between the groups were analyzed using the Chi-square test and the independent t-test. Univariate and multivariable associations between clinical variables and the outcome measures were assessed using binary logistic regression (*p* < 0.05). Candidate variables for the multivariable analysis were selected based on a univariable association with the outcome (*p* ≤ 0.20), supplemented with prespecified clinically relevant variables, irrespective of their univariable p-value. These prespecified variables included smoking habits, diabetes mellitus type II, autoimmune disease, immunosuppressive medication use, history of radiotherapy, soft tissue impaction, timing of OAC closure, surgeon’s experience and the use of an intra-alveolar hemostatic dressing. A backward stepwise selection procedure was used, sequentially removing the least contributory variables until the final multivariable model was obtained. The univariable analyses were considered exploratory and were primarily used for variable screening for the multivariable models; therefore, no formal adjustment for multiple comparisons was applied. To minimize the risk of overfitting, the number of predictors included in the multivariable logistic regression model was limited based on the commonly applied rule of approximately 10 outcome events per estimated parameter.

Statistical significance was defined as a two-tailed p-value < 0.05, and 95% confidence intervals were reported. Continuous variables were dichotomized when clinically appropriate. For variables that were included in the multivariable analysis and considered potentially modifiable, the number needed to be exposed (NNE) was calculated. The NNE was used to quantify the clinical impact of a variable of interest on a predefined outcome [24]. The NNE was derived from the experimental event rate (EER) and the control event rate (CER), representing the absolute risk reduction associated with a variable of interest. This metric estimates the number of patients who would need to be exposed to a certain variable to prevent one additional adverse outcome. A difference was made in number needed to be exposed for one additional person to benefit (NNEB) and number needed to be exposed for one additional person to be harmed (NNEH) [24]. 

## Results

**Fig. 1:**
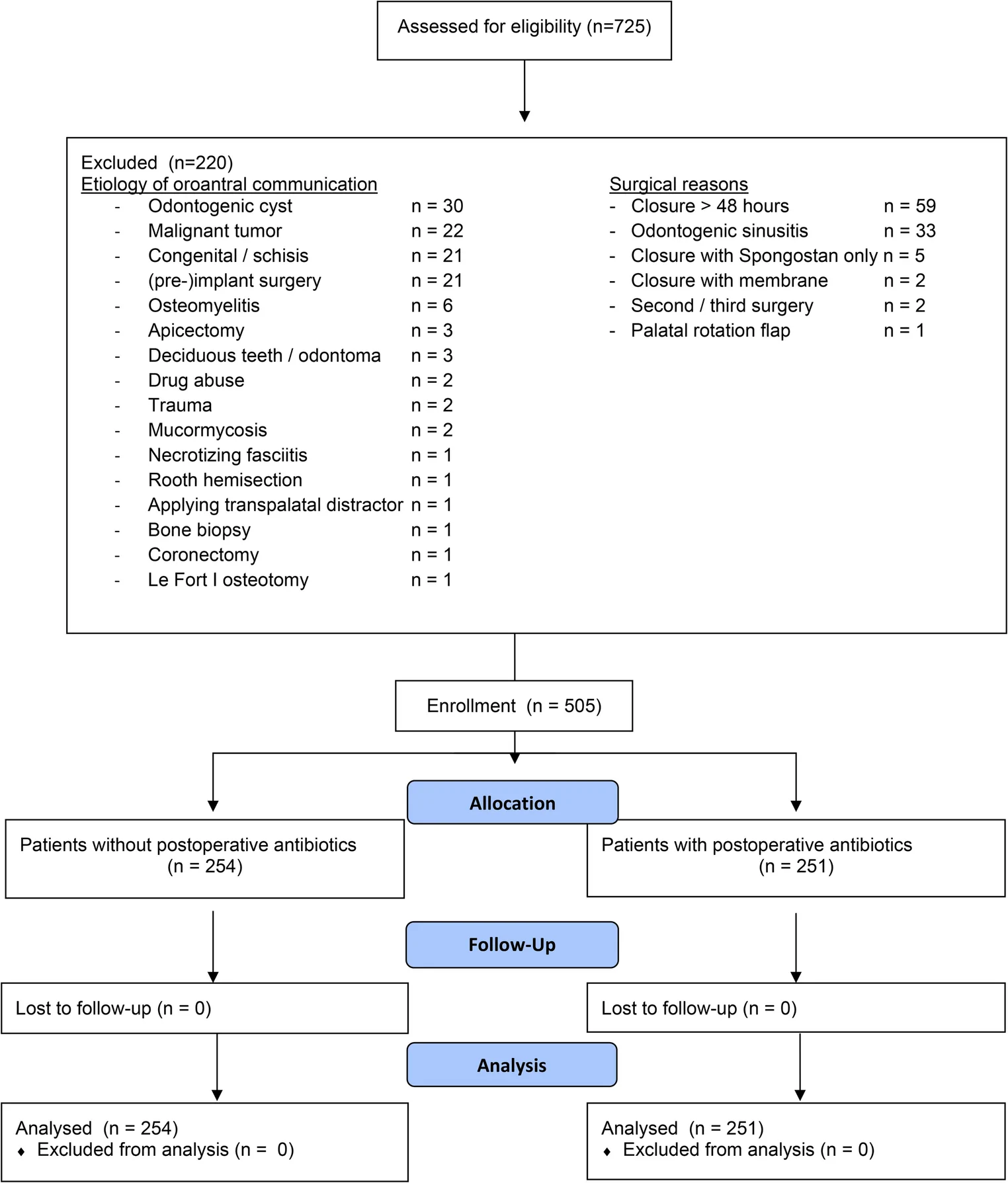
Flow diagram of included patients who underwent first-time surgical closure of an oroantral communication after tooth removal between Januari 2000 and march 2025

In total, 725 patients underwent closure of an OAC between January 2000 and March 2025. Of these, 220 patients were excluded because they did not meet the inclusion criteria, as shown in Fig. 1. Of the remaining 505 patients, 61% were male. The mean age at the time of OAC closure was 45.9 years (range 11–87). Tables 2 and 3 summarize and classify all patient characteristics according to the use of postoperative antibiotics. In total, 49.7% of the patients received postoperative antibiotics. Among these, amoxicillin–clavulanic acid was prescribed in 77.7% of cases, followed by amoxicillin (15.1%), clindamycin (4.4%), and other antibiotics (2.8%). All antibiotics were prescribed for 7 days post-operatively.

**Table 2:** Demographic and clinical characteristics of the included patients who underwent first-time surgical closure of an oroantral communication after tooth removal data are presented as number (%) unless otherwise stated

	Without post-operative antibiotics	With post-operative antibiotics	Total	*P*-value
Number of patients	254	251	505	
Age (years, mean ±SD)	44.9 (±17.3)	47.0 (±16.6)	45.9 (±17.0)	*0.17*
Sex				
Male	157 (61.8)	151 (60.2)	308 (61.0)	*0.72*
Female	97 (38.2)	100 (39.8)	197 (39.0)	
Tobacco use				
Never	197 (77.6)	205 (81.7)	402 (79.6)	*0.39*
Current	45 (17.7)	39 (15.5)	84 (16.6)	
Former	12 (4.7)	7 (2.8)	19 (3.8)	
ASA class				
Class I	128 (50.4%)	102 (40.6%)	230 (45.5%)	*0.085*
Class II	120 (47.2%)	143 (57.0%)	263 (52.1%)	
Class III	6 (2.4%)	6 (2.4%)	12 (2.4%)	
Diabetes Mellitus	22 (8.7%)	10 (4.0%)	32 (6.3%)	***0.03***
Autoimmune disease	7 (2.8%)	24 (9.6%)	31 (6.1%)	***0.001***
Antithrombotic medication	24 (9.4%)	31 (12.4%)	55 (10.9%)	*0.43*
Antiplatelet therapy	17 (6.7%)	23 (9.1%)	40 (7.9%)	
VKA or DOAC therapy	7 (2.8%)	6 (2.4%)	13 (2.6%)	
LMWH therapy	0 (0.0%)	2 (0.8%)	2 (0.4%)	
Bisphosphonate medication	1 (0.4%)	5 (2.0%)	6 (1.2%)	*0.1*
History of head and neck radiotherapy	1 (0.4%)	15 (6.0%)	16 (3.2%)	***< 0.001***
Radiotherapy dose (Gy)	NA	52.2	-	-
Affected side				
Left	141 (55.5%)	139 (55.4%)	280 (55.4%)	*0.98*
Right	113 (44.5%)	112 (44.6%)	225 (44.6%)	
Tooth type				
C	1 (0.4%)	2 (0.8%)	3 (0.6%)	*0.47*
P1	3 (1.2%)	2 (0.8%)	5 (1.0%)	
P2	12 (4.7%)	8 (3.2%)	20 (4.0%)	
M1	71 (28.0%)	88 (35.1%)	159 (31.5%)	
M2	72 (28.3%)	72 (28.7%)	144 (28.5%)	
M3	95 (37.4%)	79 (31.5%)	174 (34.5%)	
Reason for removal				
Tooth decay	68 (26.8%)	67 (26.7%)	135 (26.7%)	*0.39*
Periodontitis	26 (10.2%)	33 (13.1%)	59 (11.7%)	
Peri-apical infection	13 (5.1%)	23 (9.2%)	36 (7.1%)	
Retained root	17 (6.7%)	15 (6.0%)	32 (6.3%)	
Resorption	0 (0.0%)	1 (0.4%)	1 (0.2%)	
Impacted teeth	59 (23.2%)	51 (20.3%)	110 (21.8%)	
Fracture	16 (6.3%)	9 (3.6%)	25 (5.0%)	
Unknow	55 (21.7%)	52 (20.7%)	107 (21.2%)	

*ASA *american society of anesthesiologists, *VKA *vitamin K antagonist, *DOAC *direct oral anticoagulants, *LMWH *low molecular weight heparin, *NA *not available

**Table 3:** Radiological and surgical characteristics of the included patients who underwent first-time surgical closure of an OAC after tooth removal data are presented as number (%) unless otherwise stated

	Without post-operative antibiotics	With post-operative antibiotics	Total	P-value
Location of treatment				
VUmc	185 (72.8%)	152 (60.6%)	337 (66.7%)	***0.003***
AMC	69 (27.2%)	99 (39.4%)	168 (33.3%)	
Location where OAC occurred				
Referral / dentist	75 (29.5%)	80 (31.9%)	155 (30.7%)	*0.568*
In hospital	179 (70.5%)	171 (68.1%)	350 (69.3%)	
FROMS				
No relation	7 (2.8%)	14 (5.6%)	21 (4.2%)	*0.052*
< 1/3 root length	130 (51.2%)	132 (52.6%)	262 (51.9%)	
1/3 - 2/3 root length	83 (32.7%)	69 (27.5%)	152 (30.1%)	
2/3 root length – CEJ	14 (5.5%)	25 (10.0%)	39 (7.7%)	
Crown involvement	20 (7.9%)	11 (4.4%)	31 (6.1%)	
Soft tissue impaction				
Completely erupted	214 (84.3)	209 (83.3)	423 (83.8)	*0.659*
Partially erupted	31 (12.2)	29 (11.6)	60 (11.9)	
Non-erupted	9 (3.5)	13 (5.2)	22 (4.4)	
Timing of OAC closure				
Direct	179 (70.5%)	169 (67.3%)	348 (68.9%)	***< 0.001***
1 – 24 hours	75 (29.5%)	68 (27.1%)	143 (28.3%)	
24 – 48 hours	0 (0.0%)	14 (5.6%)	14 (2.8%)	
Type of anesthesia				
Local	241 (94.9%)	233 (92.8%)	474 (93.9%)	*0.34*
General	13 (5.1%)	18 (7.2%)	31 (6.1%)	
Surgeon’s experience				
< 1 year	59 (23.2%)	66 (26.3%)	125 (24.8%)	***0.007***
1 – 2 years	69 (27.2%)	60 (23.9%)	129 (25.5%)	
2 – 3 years	43 (16.9%)	37 (14.7%)	80 (15.8%)	
3 – 4 years	58 (22.8%)	38 (15.1%)	96 (19.0%)	
> 4 years / staff	25 (9.8%)	50 (19.9%)	75 (14.9%)	
Presence of teeth adjacent to OAC				
Edentulous	61 (24.0%)	66 (26.3%)	127 (25.1%)	*0.651*
Mesial	127 (50.0%)	123 (49.0%)	250 (49.5%)	
Distal	8 (3.1%)	12 (4.8%)	20 (4.0%)	
Mesial and distal	58 (22.8%)	50 (19.9%)	108 (21.4%)	
Fracture of the maxillary tuberosity	6 (2.4%)	8 (3.2%)	14 (2.8%)	*0.57*
Type of closure				
Buccal flap	245 (96.5%)	233 (92.8%)	478 (94.7%)	*0.07*
Buccal flap with buccal fat pad	9 (3.5%)	18 (7.2%)	27 (5.3%)	
Intra-alveolar hemostatic agent				
None	212 (83.5%)	202 (80.5%)	414 (82.0%)	*0.47*
Spongostan	41 (16.1%)	46 (18.3%)	87 (17.2%)	
Surgicel	1 (0.4%)	3 (1.2%)	4 (0.8%)	
Suture type				
Polyglactine	228 (89.8%)	213 (84.9%)	441 (87.3%)	*0.36*
Polyglactin quick	6 (2.4%)	13 (5.2%)	19 (3.8%)	
Polyglycolide	2 (0.8%)	2 (0.8%)	4 (0.8%)	
Polyglycolide quick	6 (2.4%)	5 (2.0%)	11 (2.2%)	
Unknown	12 (4.7%)	18 (7.2%)	30 (5.9%)	
Xylometazoline 0.1% nasal spray	164 (64.6%)	158 (62.9%)	322 (63.8%)	*0.71*
Chlorhexidine 0.12% mouthrinse	194 (76.4%)	177 (70.5%)	371 (73.5%)	*0.14*

*OAC* oroantral communication, *FROMS* fraction of the root overlapping the maxillary sinus, *CEJ* cementoenamel junction

Type of antibiotic was recoded as a binary variable, categorizing patients treated with amoxicillin/clavulanic acid (Augmentin) as one group and all other antibiotic regimens as the reference group. This approach was chosen because several individual antibiotic categories contained very small numbers, limiting the reliability of separate comparisons between specific antibiotic regimens. No significant differences were observed between patients who received amoxicillin/clavulanic acid (4.1%) and those receiving other antibiotic regimens (5.4%) for overall complications (*p* = 0.69). Similarly, no significant associations were found for infectious complications 1.0% vs. 0%; *p* = 1.00) and persistent OAF (1.0% vs. 1.8%; *p* = 0.53).

Patients with an autoimmune disease (*p* = 0.001) and those who received head and neck radiotherapy (*p* < 0.001) were significantly more likely to be prescribed postoperative antibiotics. Conversely, diabetic patients received antibiotics less often postoperatively (*p* = 0.03). Postoperative antibiotic use was higher at the AMC hospital than the VUmc hospital (*p* = 0.003) and in cases with delayed OAC closure was beyond 24 h (*p* < 0.001). Additionally, patients treated by surgeons with more than four years of experience received postoperative antibiotics significantly more often (*p* = 0.007).

Complications were recorded in 11.7% of the patients. The most frequent complications were wound and maxillary sinus infections (8.2%) and persistent OAFs (3.6%). Table 4 provides an overview of all complications, categorized according to the use of postoperative antibiotics. Of the 73 postoperative complications observed in this cohort, 8.2% were classified as grade I, 58.9% as grade II, and 28.8% as grade IIIa. The median time to diagnosis of a complication was 7 days (range, 0–112 days).

**Table 4:** Postoperative complications of 505 patients who underwent first-time surgical closure of an OAC after tooth removal data are presented as number (%) unless otherwise stated

Type of complication	Without post-operative antibiotics *N* = 254	With post-operative antibiotics *N* = 251	Total
Bleeding	2 (0.8)	3 (1.2)	5 (1.0)
Bone sequester	1 (0.4)	2 (0.8)	3 (0.6)
(Partial) flap necrosis	2 (0.8)	1 (0.4)	3 (0.6)
Obstructive rhinogenic maxillary sinusitis	2 (0.8)^b^	0	2 (0.4)
Persistent trismus	1 (0.4)	0	1 (0.2)
Infectious			
Local wound infection / abscess	18 (7.1)	2 (0.8)	20 (4.0)
Odontogenic maxillary sinusitis	21 (8.3)	0	21 (4.2)
Persistent oroantral fistula	15 (5.9)	3 (1.2)	18 (3.6)
Total complications	62^a^	11	73^a^
Total patients	48 (18.9)	11 (4.4)	59 (11.7)

^a^ Fourteen patients had two complications: twelve developed odontogenic maxillary sinusitis with a persistent oroantral fistula, one had flap necrosis with maxillary sinusitis

^b^ One patient developed rhinogenic sinusitis after successful OAC closure following an initial diagnosis of odontogenic sinusitis. The rhinogenic sinusitis was diagnosed 112 days after OAC closure and was subsequently treated with functional endoscopic sinus surgery (FESS). This case was unrelated to OAC closure failure

In total 3.6% (18/505) of the 505 OAC closures failed and developed a persistent OAF. In 66.7% (12/18) of these cases, maxillary sinusitis was identified as the cause, while in 33.3% (6/18) the cause was unclear. Sixteen patients with persistent OAFs received maxillary sinus irrigation (ranging from 1 to 8 irrigations), and subsequent reclosure under antibiotic prophylaxis. Two patients underwent reclosure without irrigation or antibiotics. Reclosure was performed using a buccal flap in all 18 cases, with six of these combined with a buccal fat pad (Bichat’s) flap. In three patients, a third closure using a buccal flap was required. Eventually, all OAFs were successfully closed. A Functional Endoscopic Sinus Surgery (FESS) procedure was not performed within the reclosure groups.

The mean number of follow-up consultations was significantly higher among patients who received postoperative antibiotics (1.78 ± 1.72 appointments) than among those who did not (1.27 ± 0.7 appointments) (*p* < 0.001).

Univariate analysis correlated overall complications with the use of a vitamin K antagonist (Odds Ratio (OR), 3.531; 95% Confidence Interval (CI), [1.05–11.85]; *p* = 0.041), extraction of third upper molars (OR, 1.68; CI, [1.07–3.21]; *p* = 0.027), FROMS past the cementoenamel junction (CEJ) (OR, 2.37; CI, [0.97–5.76]; *p* = 0.058), soft tissue impaction (OR, 2.14; CI, [1.14–4.03]; *p* = 0.018) and the use of postoperative antibiotics (OR, 0.19; CI, [0.10–0.39]; *p* < 0.001). Variables with a p-value ≤ 0.20 were; alendronic acid (OR, 3.88; CI, [0.69–21.65]; *p* = 0.122), root projection > 1/3 into the maxillary sinus (OR, 1.60; CI, [0.93–2.76]; *p* = 0.093), surgeon’s experience > 4 years (OR, 0.38; CI, [0.14–1.10]; *p* = 0.073), use of an intra-alveolar hemostatic agent (OR, 1.74; CI, [0.92–3.29]; *p* = 0.088), postoperative xylometazoline nasal spray (OR, 1.77; CI, [0.96–3.28]; *p* = 0.069), postoperative chlorhexidine mouthwash (OR, 1.66; CI, [0.84–3.30]; *p* = 0.148). Predefined variables were; smoking habits (OR, 0.65; CI, [0.28–1.47]; *p* = 0.299), diabetes mellitus type II (OR, 0.77; CI, [0.23–2.61]; *p* = 0.68), autoimmune disease (OR, 1.91; CI, [0.75–4.86]; *p* = 0.177), history of radiotherapy (OR, 1.08; CI, [0.24–4.89]; *p* = 0.918), immunosuppressive medication use (OR, 1.12; CI, [0.42–2.99]; *p* = 0.818), timing of OAC closure > 24 h (OR, 0.57; CI, [0.07–4.47]; *p* = 0.596), soft tissue impaction (OR, 1.20; CI, [0.35–4.20]; *p* = 0.771).

Infectious complications were correlated with extraction of third molars (OR, 2.38; CI, [1.25–4.52]; *p* < 0.008), FROMS past the CEJ (OR, 3.01; CI, [1.16–7.82]; *p* = 0.024), soft tissue (partial) impaction (OR, 2.66; CI, [1.31–5.38]; *p* = 0.007), surgeon’s level of experience > 4 years (OR, 0.13; CI, [0.02–0.97]; *p* = 0.047) and postoperative antibiotics (OR, 0.044; CI, [0.01–0.19]; *p* < 0.001). Variables with a p-value ≤ 0.20 were; male gender (OR, 1.54; CI, [0.81–2.93]; *p* = 0.184), age > 50 years(OR, 0.49; CI, [0.24–1.02]; *p* = 0.055), ASA class II and higher (OR, 0.57; CI, [0.29–1.08]; *p* = 0.085), use of anticoagulants (OR, 0.27;CI, [0.04–2.04]; *p* = 0.20), postoperative xylometazoline nasal spray (OR, 2.13; CI, [0.99–4.58]; *p* = 0.052), postoperative chlorhexidine mouthwash (OR, 1.83; CI, [0.79–4.23]; *p* = 0.158). Predefined variables were; smoking habits (OR, 0.68; CI, [0.26–1.78]; *p* = 0.429), diabetes mellitus type II (OR, 0.35; CI, [0.05–2.63]; *p* = 0.307), autoimmune disease (OR, 0.77; CI, [0.18–3.35]; *p* = 0.726), history of radiotherapy (OR, not estimable; CI, [not estimable]; *p* = 0.999), immunosuppressive medication use (OR, 0.28; CI, [0.04–2.10]; *p* = 0.215), timing of OAC closure > 24 h (OR, not estimable; CI, [not estimable]; *p* = 0.999), soft tissue impaction (OR, 0.54; CI, [0.15–1.91]; *p* = 0.340) and the use of an intra-alveolar hemostatic dressing (OR, 1.37; CI, [0.63–2.98]; *p* = 0.472). For history of radiotherapy and timing of OAC closure > 24 h, the odds ratio and corresponding confidence interval could not be reliably estimated because of the low number of events.

Persistent OAF was correlated with FROMS > 1/3 (OR, 3.46; CI, [1.21–9.85]; *p* = 0.020), soft tissue (partial) impaction (OR, 4.47; CI, [1.71–11.69]; *p* = 0.002), use of an intra-alveolar hemostatic agent (OR, 4.07; CI, [1.56–10.63]; *p* = 0.004), postoperative antibiotics (OR, 0.19; CI, [0.01–0.67]; *p* = 0.010), and postoperative chlorhexidine mouthrinse (OR, 6.39; CI, [0.84–48.47]; *p* = 0.073). Predefined variables were; smoking habits (OR, 0.29; CI, [0.04–2.18]; *p* = 0.227), diabetes mellitus type II (OR, not estimable; CI, [not estimable]; *p* = 0.998), autoimmune disease (OR, 0.90; CI, [0.12–6.96]; *p* = 0.916), history of radiotherapy (OR, 1.85; CI, [0.23–14.84]; *p* = 0.562), immunosuppressive medication use (OR, 1.52; CI, [0.34–6.87]; *p* = 0.586), timing of OAC closure > 24 h (OR, 2.15; CI, [0.27–17.35]; *p* = 0.474), soft tissue impaction (OR, 2.92; CI, [0.63–13.57]; *p* = 0.172), surgeon’s experience > 4 years (OR, 0.33; CI, [0.04–2.50]; *p* = 0.283). For diabetes mellitus type II, the odds ratio and corresponding confidence interval could not be reliably estimated because of the low number of events.

Multivariable analysis (Table 5) showed that autoimmune disease, use of an intra-alveolar hemostatic agent and postoperative antibiotic use remained significantly associated with overall complications. Infectious complications were significantly associated with soft tissue (partial) impaction and postoperative antibiotic use. Persistent OAF was significantly associated with soft tissue (partial) impaction, intra-alveolar hemostatic agent use, and postoperative antibiotic use.

**Table 5:** Multivariable regression analysis for all complications infectious complications and recurrent oroantral fistula in 505 patients with a first-time surgical closure of an OAC after tooth extractions

			CI 95%
Variable	Odds ratio	p value	Upper - Lower
All complications			
Autoimmune disease	3.597	***0.021***	1.215 - 10.649
Intra-alveolar hemostatic agent	2.058	***0.040***	1.035 - 4.093
Alendronic acid medication	5.389	*0.098*	0.735 - 39.528
Postoperative xylometazoline nasal spray	1.893	*0.052*	0.994 - 3.604
Postoperative antibiotics	0.151	***<0.001***	0.072 - 0.317
Infectious complications			
Anticoagulants	0.252	*0.192*	0.032 - 2.002
Surgeon's experience >4 years^a^	0.195	*0.125*	0.024 - 1.578
Soft tissue (partial) impaction^b^	3.892	***<0.001***	1.750 - 8.654
Postoperative xylometazoline nasal spray	2.085	*0.080*	0.915 - 4.751
Postoperative antibiotics	0.045	***<0.001***	0.011 - 0.192
Persistent oroantral fistula			
Intra-alveolar hemostatic agent	4.166	***0.008***	1.458 - 11.901
Immunosuppressive medication	3.953	*0.109*	0.735 - 21.257
Soft tissue (partial) impaction^b^	4.577	***0.003***	1.654 - 12.666
Postoperative Chlorhexidine mouthrinse	5.068	*0.112*	0.649 - 39.555
Postoperative antibiotics	0.165	***0.007***	0.044 - 0.616

*OAC *oroantral communication, *CI* confidence interval

Significant variables are highlighted in bold

^a^Surgeon’s experience categorized by experience more than 4 years

^b^Soft tissue impaction was dichotomized: fully erupted and (partial) impaction

The number NNEB for the use of postoperative antibiotics in relation to overall complications was 6.87 (95% CI: 5–11); with an absolute risk reduction of 14.6%. For infectious complications, the NNEB was 6.89 (95% CI: 5–10); with an absolute risk reduction of 14.5%. For persistent OAFs, the NNEB was 21.41 (95% CI: 13–65); with an absolute risk reduction of 4.7%. The number NNEH for the use of an intra-alveolar hemostatic agent in relation to persistent OAF was 15.0 (95% CI: 8-196); with an absolute risk increase of 6.7%.

## Discussion

Prophylactic antibiotic use following surgical closure of OAC after tooth extraction remains controversial. While some authors advocate routine postoperative antibiotic prescription, comparative evidence on postoperative outcomes is limited and largely relies on expert opinion [14–17]. The primary aim of this study was to evaluate the association between postoperative antibiotic use and overall complications, infectious complications and persistent OAF following primary surgical closure of an OAC after tooth extraction. Additionally, this study aimed to identify potential predictors of postoperative complications. We hypothesized that postoperative antibiotic use would be associated with a reduced risk of postoperative complications, particularly infectious complications.

In this multicenter retrospective cohort study, postoperative antibiotic use following primary OAC closure was associated with a significantly reduced risk of overall and infectious complications, as well as persistent OAFs. The NNEB for postoperative antibiotics in relation to overall and infectious complications was 7, indicating a potentially clinically relevant benefit. The NNEB should not be interpreted using absolute cutoff values, since its clinical relevance is context-dependent. While an ideal NNEB is 1 (every patient benefits), clinical acceptability depends on disease severity, side effect profiles, and costs.

Compared with other studies, we found a lower incidence of postoperative complications. In this series postoperative complications occurred in 11.7% of patients (18.9% without and 4.4% with postoperative antibiotics). Previously reported data indicated an overall complication rate of 31.2% following OAC closure. In these studies reported complications included partial flap necrosis (18.2%), increased granulation tissue at the suture line (11.7%), and sinusitis (7.8%). Infraorbital nerve sensory disturbances and air and/or fluid leakage each occurred in 6.5% of cases. Less frequent complications were inflammation (5.2%), flap herniation (3.9%), tooth-related flap damage (3.9%), suture dehiscence (3.9%), and herpes simplex infection (1.3%)0.2 Notably, these complications were not observed in the present study, which may be explained by the different definitions used for complications. Additionally, the present study included only patients with closure of a primary OAC.

Among 59 patients, a total of 73 postoperative complications were observed, the majority of which were infectious (41/73). Of these, 2.4% (1/41) were classified as Clavien–Dindo grade I, 56.1% (23/41) as grade II, and 41.5% (17/41) as grade IIIa. According to this information, for most patients prophylactic administration of antibiotics essentially parallels therapeutic management in terms of treatment burden. However, there is no information on patient-reported outcomes and disease burden of the complications. Additionally, literature suggests up to 10% of patients may experience gastrointestinal, allergic, or resistance-related complications [15–17]. Without this data it is difficult to advise a prophylactic or reactive antibiotic use in patients with OAC closure.

Current Dutch guidelines do not recommend routinely perioperative antibiotic prophylaxis after OAC closure following tooth extraction [12]. To the authors knowledge no international guidelines specifically address this topic. Nevertheless, despite the absence of formal recommendations, antibiotic prophylaxis appears to be frequently applied in clinical practice [8]. Similar to our study, Amoxicillin–clavulanic acid is commonly prescribed, with clindamycin used in patients with penicillin allergy [8]. In our subgroup analysis, there were no significant differences in postoperative outcomes between patients who received amoxicillin/clavulanic acid and those receiving other antibiotic regimens. Therefore, our findings do not provide evidence of a significant difference in postoperative outcomes between these antibiotic regimens.

Currently, only one study evaluated prophylactic antibiotic use prior to OAC closure following tooth extraction. In this study, 22 of 46 patients received a single preoperative dose of 2 g amoxicillin, while 24 patients did not receive antibiotic prophylaxis. No postoperative complications were observed in the antibiotic group, whereas 12.5% (3/24) of patients without prophylaxis developed postoperative sinusitis [25]. The study suggested that prophylactic antibiotics may not be indicated for preventing postoperative complications, such as sinusitis, following closure of oroantral perforations. However, it stated that confirmation through prospective randomized trials is warranted. While the incidence of postoperative sinusitis appeared lower in the antibiotic group, the limited sample size may have reduced the statistical power to detect a clinically relevant difference between the groups. Direct comparison of these data with our study is challenging. Nevertheless, the observed effectiveness may be related to the antibiotic regimen and could potentially support a longer duration of antibiotic therapy. Conversely, other studies reported that short-course antibiotic prophylaxis (≤ 24 h) is as effective as prolonged regimens (≥ 72 h) in preventing surgical site infections in Ear, Nose, Throat (ENT) and Oral and Maxillofacial surgery, while longer courses significantly increase the risk of adverse events [26]. 

Soft tissue impaction of teeth was associated with infectious complications and persistent OAFs in this study. Several mechanisms may underlie this association. Bacterial accumulation beneath gingival flaps can lead to local infection (pericoronitis), thereby compromising tissue quality, impairing healing capacity, and facilitating contamination of the surgical site [27]. In addition, a previous study indicated that both the condition and the quantity of gingival tissue are critical determinants of successful oroantral communication (OAC) closure, underscoring the increased risk associated with soft tissue–impacted teeth, which may help explain the increased risk observed in association with soft tissue–impacted teeth [4]. 

The observed association between autoimmune disease and overall complications may be explained by several mechanisms. Patients with autoimmune disorders may have an altered systemic inflammatory and immune response, which could impair the normal inflammatory and reparative processes involved in postoperative wound healing. In addition, autoimmune diseases are frequently treated with immunomodulatory or immunosuppressive medication, which may further affect host defense and tissue repair and thereby increase susceptibility to postoperative infection or impaired wound healing. Furthermore, autoimmune disease may be accompanied by systemic factors such as chronic inflammation and vascular or connective tissue abnormalities, which could potentially compromise tissue regeneration [28]. 

The use of an intra-alveolar hemostatic agent was associated with an increased risk of overall complications and persistent OAFs. It is important to note that in all cases in this study, the OAC was not managed with a hemostatic agent alone, but were surgically closed with placement of the agent beneath a local flap. A possible explanation for this finding could be a foreign -body effect, facilitating microbial adherence and subsequent infection. Comparative studies of gelatin sponge (Spongostan) and oxidized regenerated cellulose (Surgicel) in post-extraction sockets demonstrate no significant differences in postoperative bleeding between sockets treated with hemostatic materials and those left to heal without them [29]. In contrast, the use of these materials is associated with increased postoperative pain and swelling, with pain being more pronounced in the gelatin sponge group and a slight delay in wound healing observed [29]. Similarly, another study concluded that the use of oxidized regenerated cellulose in open prostatectomy was ineffective in controlling bleeding and may increase the risk of infection [30]. 

The limitations of this study are inherent to its retrospective design and the variability in clinical assessment and surgical practice. In procedures performed under local anesthesia, the presence of an OAC was assessed using a Valsalva maneuver, whereas in procedures performed under general anesthesia, the surgeon may have used probing or visual inspection alone. This variability in OAC assessment may have resulted in differential detection of OACs and may have affected the internal validity of the study.

Although the data may be considered reflective of real-world practice, confounding by indication in antibiotic prescribing cannot be excluded. This is highlighted in the baseline statistics as there are significant differences between the groups who received postoperative antibiotics and those who did not. Notably, factors theoretically associated with an increased risk of postoperative complications after OAC closure were more prevalent in the antibiotic group, which highlight the potential protective effect of postoperative antibiotic therapy on clinical outcomes. Furthermore, variability among surgeons and centers may have influenced both treatment selection and postoperative outcomes. This may have resulted in residual confounding and complicated the interpretation of the observed association between antibiotic use and postoperative complications. This may be reflected in the counterintuitive findings for chlorhexidine mouthrinse and xylometazoline nasal spray, both of which appeared to be associated with an increased risk of postoperative complications. However, neither variable reached statistical significance in the multivariable analysis. One possible explanation is that these medications were more frequently prescribed in patients with more complex or challenging cases, suggesting the presence of confounding by indication and further highlighting the potential for residual confounding. Furthermore the multiple univariable analyses may have increased the risk of type I error, although these analyses were primarily used for exploratory variable screening rather than confirmatory inference.

The use of a backward stepwise selection procedure may have introduced model-selection bias, as the selection of variables was data-driven and may result in instability of the estimated associations. This should be considered when interpreting the multivariable findings.

Finally, antibiotic-related adverse events were not systematically recorded in this study and could therefore not be assessed. Consequently, potential adverse events associated with antibiotic use, such as gastrointestinal or allergic reactions, as well as the broader concern of antimicrobial resistance, could not be directly weighed against the observed reduction in postoperative complications.

A strength of this study is the inclusion of a well-defined multicenter cohort.

Prospective studies are needed to better assess confounding and better define which patients could benefit from perioperative antibiotics. Incorporating patient-reported outcomes related to complications and treatments, systematic assessment of antibiotic-related adverse events, and microbiologically guided treatment strategies could further clarify the role of antibiotics in OAC management and support evidence-based guidelines.

## Conclusion

In this multicenter retrospective cohort study, postoperative antibiotic use after primary OAC closure following tooth extraction was associated with a reduced risk of overall and infectious complications and fewer persistent OAFs. The observed absolute and relative risk reductions, together with the number needed to be exposed suggest a clinically relevant benefit. Nevertheless, routine antibiotic prescription cannot be recommended, as these results should be confirmed with prospective randomized trials. Furthermore, data on patient-reported outcomes, outcome severity, and treatment burden are lacking. Given that most complications were managed conservatively or pharmacologically, these findings support a selective, individualized approach to postoperative antibiotic use. 

## Data Availability

The data that support the findings of this study are available from Amsterdam University Medical Centers (Amsterdam UMC). However, restrictions apply to the availability of these data, which were used under institutional permission for the current study and are therefore not publicly available. The data may be available from the corresponding author upon reasonable request and with the permission of Amsterdam UMC.
